# 191. When *Staph* Is Not Solo: Polymicrobial Methicillin-Resistant *Staphylococcus aureu*s Bloodstream Infections

**DOI:** 10.1093/ofid/ofad500.264

**Published:** 2023-11-27

**Authors:** Angelica Escalona, Emi Hayashi, Michelle Evans, Harm van Bakel, Amy C Dupper, Bremy Alburquerque, Russell B McBride, Deena Altman

**Affiliations:** Icahn School of Medicine at Mount Sinai, New York, New York; Icahn School of Medicine at Mount Sinai, New York, New York; Icahn School of Medicine at Mount Sinai, New York, New York; Icahn School of Medicine at Mount Sinai, New York, New York; Icahn School of Medicine at Mount Sinai, New York, New York; Icahn School of Medicine at Mount Sinai, New York, New York; Icahn School of Medicine at Mount Sinai, New York, New York; Icahn School of Medicine at Mount Sinai, New York, New York

## Abstract

**Background:**

Methicillin-resistant *Staphylococcus aureus* (MRSA) bloodstream infection (BSI) is a serious public health concern. At times, MRSA is isolated from the blood along with other pathogens, the significance and the consequences of which are not well described. This study aims to outline the clinical characteristics and outcomes of those with polymicrobial MRSA BSI compared with those with monomicrobial MRSA BSI.

**Methods:**

We conducted a retrospective case-control study of those with and without polymicrobial MRSA BSI from 2014 to 2022 at a single quaternary care center in New York. Risk factors and outcomes for polymicrobial MRSA BSI were assessed using logistic regression analyses in SAS.

**Results:**

Of 646 patients with MRSA BSI during the study period, 48 (7.4%) had polymicrobial MRSA BSI. In the univariate analysis, the presence of urinary device (*p* = 0.0174), and gastrointestinal device (*p* = 0.0287) were significantly associated with polymicrobial BSI. Polymicrobial BSI was associated with ICU admission after BSI (*p* = 0.0184). Mortality did not differ. Gram-positive *Enterococcus* sp. (22%) was the most common co-pathogen of MRSA in polymicrobial BSI.

**Conclusion:**

While polymicrobial BSI remains relatively infrequent, risk factors for development may include devices in the genitourinary and gastrointestinal systems. Those who develop polymicrobial MRSA BSI may be at higher risk of being admitted to the ICU, signifying increased morbidity during hospital stay. Further studies are needed to evaluate the impact of polymicrobial MRSA BSI to elucidate the interplay between pathogens and the impact on infected hosts.

**Disclosures:**

**All Authors**: No reported disclosures

